# Probiotics as a Potential Alternative for Relieving Peripheral Neuropathies: a Case for Guillain-Barré Syndrome

**DOI:** 10.3389/fmicb.2015.01497

**Published:** 2016-01-07

**Authors:** Abhishek Saxena

**Affiliations:** Department of Biotechnology, TERI UniversityNew Delhi, India

**Keywords:** Guillain–Barré syndrome, peripheral neuropathies, neuronal autoimmunity, probiotics, microbiota, gut–brain axis

## Introduction

Trillions of microorganisms form the “natural flora” of the human body. These microorganisms outnumber the host cells 10 to 1, essentially making humans a microbial ecosystem of sorts. Before the advent of the high-throughput sequencing technologies such elusive microorganisms were uncultivable and could not be studied using traditional microbiological techniques. However, since the past decade, with the establishment of the Human Microbiome Project reference database in 2012, research in this area has grown logarithmically (Grogan, 2015). The human microbiota is introduced from mother to the fetus and even infants, and remains with the growing individual for the rest of his/her life, being effected by and also affecting the individual's lifestyle, food habits, and metabolism. Friendly microbes have been shown to protect from several diseases and a dysbiosis in the microbiota has been linked to infections by pathogens, autoimmunity and lifestyle disorders. The establishment of the brain-gut axis (reviewed in Carabotti et al., 2015) in the past few years has even shown that gut microbiota and brain affect each other (Carabotti et al., 2015). A very recent study established the missing link between the central nervous system (CNS) and the immune system i.e., the CNS lymphatic vessels, thus completing the microbiota-neuronal-immune system triangle (Louveau et al., 2015). Even stress, and behavioral changes can result in an altered microbiota and vice–versa (Schmidt, 2015). This has brought microbiologists and neuroscientists together to look into the probable mechanisms in which dysbiosis of gut microbiota may affect development of neurodegenerative diseases and even neuronal autoimmunity.

Autoimmune diseases of both the central and the peripheral nervous system (PNS) have been linked to microbiota, although the extent to which the link has been established is very different. While some like multiple sclerosis (MS) has working mice-models, others like Guillain–Barré syndrome (GBS), a rare autoimmune disorder of the PNS has no defined models, although experimental autoimmune neuritis (EAN) may be used to study some aspects of GBS, especially macrophage mediated demyelination (Kieseier et al., 2012). Even so, we have been warned against the fallacies of studying such models in isolation, as there is no strict one model-one disorder kind of relationship between human autoimmunity and mouse/rat model (Gold et al., 2000). Since probiotics have been found to be useful in the treatment of autoimmune diseases like Inflammatory Bowel Disease (IBD) (Sheil et al., 2007), type1 diabetes (Calcinaro et al., 2005) and even extra-intestinal neuronal and systemic-autoimmune disorders like MS (Lavasani et al., 2010) and systemic lupus erythematosus (SLE) (Manirarora et al., 2008; Alard et al., 2009), testing the efficacy of probiotics in the treatment of GBS might offer a less invasive and more acceptable and economical treatment alternative.

## Probiotics modulate the gut microbiota to affect the host immune system

Altered gut microbiota or “gut dysbiosis” has been linked to autoimmune responses both intra-intestinally as well as extra-intestinally. Recently concluded and active research has helped detail the mechanisms of interaction between the gut microbiota and the immune system (Maynard et al., 2012). Attempts to understand the mechanisms of restoration of healthy gut microbiota (eubiosis) by probiotics has started to reveal the complex interactions and signaling involved between the host cells including components of the immune system, the probiotic strain and the other commensal or pathogen residing in the host system (Wagner et al., 2009; Wine et al., 2009; Maynard et al., 2012; Bollrath and Powrie, 2013). It is generally accepted that adaptive immunity to microbes may be mediated by Toll-like receptors (TLRs), C-type lectin receptors (CLRs), and NOD-like receptors (NLRs), which are present on the epithelium, endothelium, and lymphoid tissue of the host. These receptors might signal downstream depending upon the situation of eubiosis (homeostasis)/dysbiosis (Walker, 2008; Maynard et al., 2012). Traditionally, two T-cell fates have dominated the immune system related signaling *viz.* T-helper cell (T_H_1) and T_H_2. T_H_1 type response is elicited on exposure to pathogens and leads to their phagocytosis, causing inflammation to the surrounding host tissue and even autoimmune responses. T_H_2 type of response is elicited mainly on exposure to environmental cues and also some pathogenic proteins, leading to the production of IgE and release of histamines that counter T_H_1 response (Berger, 2000). Later studies have, however, revealed a much more complex picture of autoimmunity and has introduced new types of CD4^+^ T-cell *viz.* T_H_17 (Damsker et al., 2010) and CD25^+^ FoxP3^+^ regulatory T-cells (Treg) (Sakaguchi et al., 2009). During immune homeostasis the concentration of Interleukin (IL-10) (also secreted by T_H_2 cells) secreting Treg cells dominate the intestinal mucosa. In most of the autoimmune conditions, a common feature seems to be diminished presence of Treg cells in the intestinal mucosa of the host resulting in a shift toward IL-17 secreting T_H_17 cells and this shift in the T-cell population seems to be dependent on the intestinal cytokine Transforming Growth Factor (TGF-β). Interestingly, in the face of pathogenic infection or an inflammation caused by the resident microbiota, the IL-10 secreting Treg cells are also converted to IL-17, Retinoic acid-related Orphan Receptor (RORγT), and Interferon (IFN-γ) producing T_H_17 cells (Xu et al., 2007) and vice–versa (Gagliani et al., 2015). Research exploring the gut-brain axis has lead to an understanding that altered gut microbiota plays a role not only in gastrointestinal autoimmunity but also extra-intestinal autoimmunity. Systemic and neuro-autoimmunities like SLE and MS have been linked to gut dysbiosis, making a strong case for linking GBS to altered microbiota.

Our current understanding of the link between gut eubiosis and immune homeostasis, and the effect of probiotics on the former have been reviewed elsewhere (Hemarajata and Versalovic, 2013; Kueffel et al., 2013). Various studies have already tipped the balance toward the use of probiotics for providing relief in a number of diseases and disorders including extra-intestinal autoimmunities. As briefly mentioned above, a study has pointed toward the therapeutic effect of probiotic consisting of a mixture of *lactobacilli* on experimental autoimmune encephalomyelitis (EAE; an experimental model of MS) through IL-10 secreting Treg cells (Lavasani et al., 2010). In a later study, it has been shown that pro-inflammatory T-cell response consisting of IL-17A and IFN-γ producing T_H_17 cells caused EAE in germ-free mice upon infection from Segmented Filamentous Bactria (SFB) (Lee et al., 2011), further linking gut dysbiosis and neuronal autoimmunity. Immunopathology of GBS too is complicated by T_H_17 cells and the corresponding cytokines, thus making it a balance between T_H_1/T_H_17 and T_H_2, although mechanistic details are yet to be elucidated (Liang et al., 2012; Li et al., 2012, 2014). Functional aspects of T_H_17 cells and associated cytokines and their rational drug targeting with respect to GBS is discussed in some details elsewhere (Wang et al., 2013; Wu et al., 2015). Studies on the lines of drug trials have shown that in most stomach related ailments; probiotics do confer a health benefit (Mack, 2005). Some small studies in model organisms show that probiotics confer prophylactic or curative benefit against diabetes, certain cancers, Human Immunodeficiency Virus (HIV), rheumatoid arthritis, and MS among several other conditions (Erickson and Hubbard, 2000; Matsuzaki et al., 2007).

## Guillain–Barré syndrome: prototype of an immune mediated peripheral neuropathy

GBS is a post-infection autoimmune monophasic disorder of the PNS and is characterized by acute flaccid paralysis. The syndrome has variants categorized depending upon the kind of neuropathy *viz.* demyelinating or axon degenerating. These have been described in detail by Dimachkie and Barohn (2013) and have been summarized in the form of a table in this article (Table 1). The immunopathology of the GBS is not well understood however it is known that the syndrome occurs post-infection by *Campylobacter jejuni* (Kaldor and Speed, 1984; Allos, 1997), *Mycoplasma pneumonia* (Goldschmidt et al., 1980), certain Herpesviridae (Jacobs et al., 1998), HIV (Pontali et al., 2011) and flu viruses (Sivadon-Tardy et al., 2009) or in some cases after the administration of flu vaccine (Geier et al., 2003), most probably due to an imbalance in the various T-helper cell populations described above. Proinflammatory, T_H_1 type cytokines like Tumor Necrosis Factor (TNF-α), IFN-γ, and IL-1 have implicated in triggering mechanism of EAN/GBS. Other proinflammatory cytokines like IL-12, TNF-α/β, and IL-2 are also detected during the course of the disease progression. T_H_17 cytokines IL-17 and IL-22 have also been detected in the serum of GBS patients thus confirming the role of T_H_17 mediated pro-inflammatory response (Li et al., 2012). Interestingly, proinflammatory cytokines IL-4, IL-5, and IL-6, may be produced by cells expressing the rival T_H_2 phenotype, contributing to GBS. However, T_H_2 type cytokines IL-10 and TGF-β are associated with receding EAN/GBS symptoms (Zhu et al., 1998). Both T-cell mediated and humoral immunity seems to be playing a role in GBS. In Acute Inflammatory Demyelinating Polyneuropathy (AIDP) variant, activated T-cells, upon antigen presentation by macrophages, cross the blood-nerve barrier (BNB) and release cytokines that activate endoneural macrophages, damaging the compact myelin. Alternatively either by directly recognizing a pathogenic epitope or elicited by T-cell, B-cells produce antibodies to the cross-reactive axolemmal autoantigens and by fixing a complement leads to the damage of Schwann cells and/or axons, leading to Acute Motor Axonal Neuropathy (AMAN), the more severe Acute Motor and Sensory Axonal Neuropathy (AMSAN), or Miller Fisher Syndrome (MFS) (Hughes and Cornblath, 2005). The most common autoantigens are GM1, GM1a, and GD1a (Dimachkie and Barohn, 2013). Due to these variants and their diverse pathology and pathogenesis, GBS is now considered a group of heterogeneous conditions with similar clinical phenotypes (Kieseier et al., 2012). GBS has been considered a unique autoimmune disorder due to the fact that unlike most other neuronal autoimmune disorders, it is monophasic and that its occurrence has been found to correspond to immunosuppression in the patient (Steiner et al., 2010). The antecedent causative organisms mostly linked with GBS include *C. jejuni* (≈38%) (Kaldor and Speed, 1984), *M. pneumonia* (≈21%) (Sharma et al., 2011). Infections by most or all of these agents are characterized by immunosuppression either as a primary result of the infection or as a secondary effect.

**Table 1:** **Variants of Guillain–Barré Syndrome**.

**GBS variant**	**Symptoms**	**Mechanism of autoimmunity**	**Autoantigens implicated**	**Remarks/references**
AIDP	Areflexia, mild sensory changes, distal paresthesisas, loss of tendon reflexes, ascending paralysis, respiratory failure	Macrophages, T-cell mediated demyelination		Dimachkie and Barohn, 2013
AMSAN	Loss of deep tendon reflex, distal weakness and sensory aymptoms	Auto antibodies against nerve gangliosides	GD1a, GM1, GM1b	Shoenfeld and Meroni, 2012; Dimachkie and Barohn, 2013
AMAN	Accute, flaccid ascending paralysis, high protein in the CSF, dysphagia, dysarthria, total loss of refexes, and respiratory failure in advanced cases	Auto antibodies against nerve gangliosides	GD1a, GM1, GM1b, GalNac-GD1a	Discovered in China, less common than AIDP in the West (Shoenfeld and Meroni, 2012; Dimachkie and Barohn, 2013)
MFS	Ophthalmoplegia, ataxia, areflexia	Auto antibodies against nerve gangliosides	GQ1b	5–10% cases in West but up to 25% in Japan (Shoenfeld and Meroni, 2012; Dimachkie and Barohn, 2013)

## Eubiotic gut to cure Guillain-Barré syndrome

Infection by *C. jejuni, M. pneumonia*, and viruses mentioned above also shows a differential effect on Treg and T_H_1 cell populations with infected individuals showing a reduced Treg cell presence (Steiner et al., 2010). Besides, there is evidence of *C. jejuni* colonization being exacerbated by a shift in the microbiota (characterized by increased *Escherichia coli* load in the gut) upon infection by *Toxolasma gondii* (Haag et al., 2012) and its alleviation by probiotic strain *Lactobacillus helveticus* strain R0052 (Wine et al., 2009). With studies pointing toward probiotic assisted increase in the population of anti-inflammatory Treg cells (Smits et al., 2005; Kwon et al., 2010), and probiotics modulating T_H_1(and T_H_17)/T_H_2 balance (Torii et al., 2007; Tanabe, 2013) it may be worth testing whether this increase in Treg cells reduces post infection autoimmunity in any way. It may not be wrong to expect a Treg cell mediated immune homeostasis of both the humoral (Wing and Sakaguchi, 2014) and cellular kind (Shevach, 2009), produced as a result of probiotic induced gut eubiosis.

## Conclusion

GBS is a rare autoimmune disease effecting 2–4 people per 100,000. Being a rare condition it is neglected by the big pharmaceutical companies, as well as academic researchers searching for better treatments. Although the treatments are available in the form of intravenous immunoglobulin (IVIg) administration and plasma exchange, they become quite expensive, especially in the developing countries. Added to it the poor prognosis of the disease, there is a need for cheaper alternatives to the currently available treatments. Probiotics have been shown to be effective in treatment of several intestinal and extra-intestinal autoimmunities, as they act by replenishing the Treg cells in the immune system that have been shown to promote the immune system homeostasis. There is ample reason to believe that GBS also is a result of altered immune homeostasis caused by infection due to an antecedent infection. Thus, promoting the production of Treg cells may help cure GBS by acting against both T- cell mediated and humoral autoimmunity of the PNS.

## Author contributions

The author AS claims the sole responsibility of researching and writing this article.

### Conflict of interest statement

The author declares that the research was conducted in the absence of any commercial or financial relationships that could be construed as a potential conflict of interest.
